# Alcohol dehydrogenase and aldehyde dehydrogenase in malignant neoplasms

**DOI:** 10.1007/s10238-016-0408-3

**Published:** 2016-02-17

**Authors:** Karolina Orywal, Maciej Szmitkowski

**Affiliations:** Department of Biochemical Diagnostics, Medical University of Bialystok, Waszyngtona 15 A, 15–276 Białystok, Poland

**Keywords:** Alcohol dehydrogenase isoenzymes, Aldehyde dehydrogenase, Cancer diseases, Alcohol metabolism

## Abstract

According to International Agency for Research on Cancer, ethanol and acetaldehyde belong to group 1 of human carcinogens. The accurate mechanism by which alcohol consumption enhances carcinogenesis is still unexplained. Alcohol is oxidized primarily by alcohol dehydrogenase (ADH) to acetaldehyde, a substance capable of initiating carcinogenesis by forming adducts with proteins and DNA and causing mutations. Next, acetaldehyde is metabolized by aldehyde dehydrogenase (ALDH) to acetate. In tissues of many cancers, we can observe significantly higher activity of total alcohol dehydrogenase with any change in aldehyde dehydrogenase activity in comparison with healthy cells. Moreover, in malignant diseases of digestive system, significantly increased activity of ADH isoenzymes class I, III and IV was found. The gynecological, brain and renal cancers exhibit increased activity of class I ADH. ADH and ALDH can play also a crucial regulatory role in initiation and progression of malignant diseases by participation in retinoic acid synthesis and elimination of toxic acetaldehyde. Besides, changes of enzymes activities in tumor cells are reflected in serum of cancer patients, which create the possibilities of application ADH isoenzymes as cancer markers.

## Introduction

Alcohol ingestion is widespread throughout the world. Although ethanol consumption is commonly accepted as social activity, it is estimated that it is responsible for 3.8 % of global mortality and for 6.5 % of deaths in Europe [1]. It has been known that alcohol abuse is associated with damaging effects on the central and peripheral nervous system, liver and pancreas or myocardium. A great number of epidemiological studies have demonstrated a correlation between ethanol consumption and the occurrence of cancers in many organs. Worldwide, about 3.6 % of all cancers were attributable to alcohol drinking and the International Agency for Research on Cancer (IARC) classified alcohol consumption as group 1 of human carcinogens [2]. A link has been established between alcohol and cancers of oral cavity, pharynx, larynx, esophagus, stomach, liver, pancreas, colon, rectum and breast.

There is a dose–response relationship between ethanol and cancer risk, with results showing that the risk of cancer increases with increasing amount of alcohol consumed [3]. The strongest association is observed for drinking, in particular regular heavy drinking. Moreover, there is no consistent difference in cancer risk between different types of alcoholic beverages [4]. The average consumption of alcohol in Europe is more than twice the global average, and the average number of alcohol-attributable cancer also exceeds by far the global average [5]. There is a strong recommendation to limit consumption of alcoholic drinks to less than 20 g per day for men and 10 g for women which leads to decreasing the risk of cancer about 6 % [6].

Ethanol is absorbed by the small intestine and metabolized by liver in the three ways: oxidation by alcohol dehydrogenase (ADH), cytochrome P450 2E1 and catalase. These reactions lead to form acetaldehyde, which is converted into acetate by aldehyde dehydrogenase (ALDH). The main participation in alcohol metabolism accounts for alcohol dehydrogenase. Alcohol metabolism with ADH leads to the generation of reduced forms of nicotinamide adenine dinucleotide (NADH), but induction of cytochrome P450 2E1 conveys to reactive oxygen species production. Acetaldehyde and reactive oxygen species combine with cell compounds which causes disturbances of cell physiological functions. Human ADH and ALDH exist in multiple molecular forms that have been grouped into several classes, and the different isoenzymes have different catalytic properties.

Many studies have shown that disturbances of the alcohol dehydrogenase activity play an important role in alcohol-related neoplasms, and knowledge of possible mechanism by which alcohol effects on carcinogenesis has increased in recent years.

## Mechanism of alcohol carcinogenicity

**Fig. 1:**
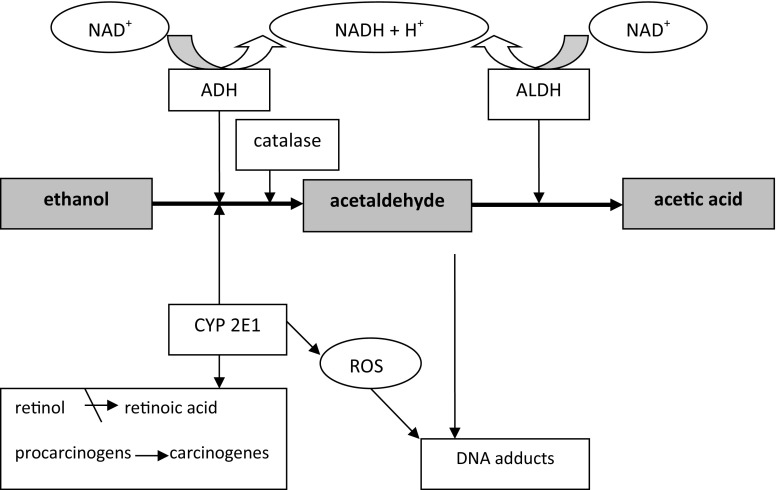
Metabolism of alcohol and its simplified role in carcinogenesis

There are a number of biological mechanisms that may explain the link between alcohol consumption and cancer development (Fig. 1). One theory is that exposure to high levels of acetaldehyde is associated with alcohol-related cancer. Acetaldehyde causes mutations and sister chromatid exchanges in human cells [7]. It interferes at many sites with DNA synthesis and repair, which results in tumor development [8]. Moreover, acetaldehyde inhibits *O* ^6^-methyl-guanyltransferase, an enzyme necessary for reactivation of adducts caused by alkylating agents [9]. Acetaldehyde, by binding to DNA and cellular protein, forms adducts, which play an important role in carcinogenesis [10]. DNA adducts, such as *N* ^2^-ethylidene-2′-deoxyguanosine, *N* ^2^-ethyl-2′-deoxyguanosine and 1,*N* ^2^-propano-2′-deoxyguanosine, cause polymerase errors and induce mutations in critical genes. Moreover, they can lead to activation of proto-oncogenes, inactivation of tumor suppressor genes in replicating cells and inhibition of many important enzymes of DNA synthesis pathways [11]. The formation of DNA adducts can be facilitated in the presence of amino acids, histones or polyamines, which increased concentration we can find in tissues with hyper-regeneration. Chronic alcohol consumption results in induction of inflammation and metaplasia of epithelium, causing cell damage and consequently hyper-regeneration of tissues [12].

Chronic alcohol consumption causes the activation of microsomal ethanol oxidizing system (MEOS) and expression of CYP 2E1. In humans, the induction of CYP 2E1 increases 10–20 times after daily ingestion of 40 g of ethanol just for 1 week [13]. Induction of CYP 2E1 expression is one of the central pathways by which ethanol generates production of reactive oxygen species (ROS) [8]. Moreover, an important consequence of ethanol and acetaldehyde oxidation is an increase in both cytosolic and mitochondrial NADH/NAD ratios, which elevate the activity of xanthine oxidase, a free-radical generating enzyme. It is commonly known that ROS can cause oxidative damage to proteins, lipids and nucleic acids. The most reactive ROS are: superoxide, hydrogen peroxide, hydroxyl radicals and singlet oxygen. These compounds can cause DNA damage by oxidation bases, making breaks in single and double strand and also by generating oxidative DNA adducts [9]. Moreover, ROS influence on lipids in cell membranes causing their peroxidation. The end products of lipid peroxidation may damage DNA through the formation of exocyclic adducts. The main products of lipid peroxidation are: crotonaldehyde, acrolein, 4-hydroxy-2-nonenal and malondialdehyde [14]. DNA modified by peroxidation products is genetically unstable. Moreover, CYP 2E1 metabolizes various procarcinogens present in diets and tobacco smoke to their carcinogenic metabolites [15].

CYP 2E1 also degrades retinoic acid (RA) and retinol to polar metabolites with toxic and apoptotic properties which can promote carcinogenesis. Chronic alcohol consumption affects also the additional aspects of vitamin A metabolism, including retinol absorption, enhanced degradation in liver and increased mobilization of retinol from the liver to the other organs [16]. Furthermore, it has been demonstrated that ethanol acts as a competitive inhibitor of retinol oxidation in liver which may reduce the biosynthesis of retinoic acid [17]. These alcohol-induced changes result in decreasing hepatic levels of retinol, precursor of RA, the most active form of vitamin A. Retinoic acid is particularly important because of its effect on cellular growth, differentiation and apoptosis. Tissues depleted of retinoic acid may enter a premalignant state, characterized by low differentiation and high degeneration. Thus, disturbances in RA biosynthesis may provide a possible explanation for why chronic and excessive alcohol intake is a risk for cell proliferation and malignant transformation. Chronic alcohol consumption may lead also to nutritional deficiencies (vitamin A, vitamin C, zinc, iron and folate) by impaired intestinal absorption and by changes in metabolic pathways. The most visible effect seems to be on folate, vitamin B12 and vitamin B6 metabolism, resulting in further changes in DNA-methylation pathways [18]. Inhibition of trans-methylation results in disturbances in the activity of genes entangled in carcinogenesis.

Alcohol-related cancers are difficult to cure because ethanol consumption influences chemotherapeutic agent metabolism which results in a decreasing response to medication and increase in side effects. Moreover, during cancer development, alcohol consumption may contribute to inflammatory and immunosuppressive environments, thus allowing tumor cells to propagate and spread [20]. Ethanol can also accelerate tumor growth and stimulates progression probably due to induction of angiogenesis. Alcohol consumption causes up-regulation of the expression of vascular endothelial growth factor (VEGF) which affects the increase in both tumor size and intratumoral vascular volume density [19]. Furthermore, alcohol may affect the stem cell niche by perturbing many biochemical or signaling pathways (prolactin/growth hormone (GH), the IGF-1, estrogen receptor (ER), TGF-β, integrins, telomerase) known to be important for both stem cells and cancers. Alcohol and its metabolites may interfere with the renewal or differentiation of stem cells or cancer stem cells by affecting one or more of these pathways [20].

## Alcohol dehydrogenase and aldehyde dehydrogenase

The major pathway for the elimination of ethanol is its oxidation to acetaldehyde by alcohol dehydrogenase (ADH). Acetaldehyde transforms to acetate in the reaction catalyzed by aldehyde dehydrogenase (ALDH). Mammalian ADH and ALDH constitute a complex enzyme family, expressed in a few molecular forms, grouped into several classes, which differ by localization, substrate specificity, catalytic and immunological properties.

The class I isoenzymes are dimers formed with *α,* *β* and *γ* polypeptide subunits, encoded by three separate gene loci: *ADH1A*, *ADH1B* and *ADH1C*. Genetic polymorphism occurs in the class I isoenzymes among racial populations, which affects the acetaldehyde production. In Asians, polymorphism of *ADH1B* and *ADH1C* genes was associated with the development of alcoholism and susceptibility to alcoholic liver cirrhosis [21]. ADH I is expressed mostly in the liver but also can be found in the lungs, kidneys and gastrointestinal tract (duodenum and colon) [22]. Class II ADH is a homodimer (*π* *π*) encoded by *ADH2* which exists only in the liver [23]. Class III alcohol dehydrogenase (*χ* *χ*) is found in every tissue and is encoded by *ADH3* loci. ADH III has the same structure and kinetic properties as glutathione-dependent formaldehyde dehydrogenase [24]. Because of its localization and very high activity in stomach, class IV ADH has been termed “gastric” alcohol dehydrogenase. It is a homodimer (*σσ* or *μμ*) encoded by *ADH4* loci. Besides stomach, this isoenzyme is found also in esophagus, liver, skin and cornea, but its expression is limited [25]. The other classes of ADH are still poorly described. ADH V is a homodimer, encoded by *ADH5*, which was found in liver and epithelium of stomach mucosa membrane [26]. Class VI is expressed in liver and kidneys of rats and draws high analogy with mammalian ADH V (67 %) [27].

Ethanol is the main substrate for alcohol dehydrogenase, but its oxidation is efficient only in reaction catalyzed by isoenzymes class I and IV. Besides alcohol, ADH I takes part in metabolism of many aldehydes, bioamines, prostaglandins, steroids and *ω* fatty acids [28]. Moreover, this class participates in oxidation of retinol to retinal, a substrate for retinoic acid biosynthesis [29]. ADH II catalyzes oxidation of ethanol but mostly in the presence of its high concentrations. It takes part also in retinal production, but because of its limited localization, it catalyzes synthesis of retinol only in the liver [30]. Isoenzymes of class III reveal a high affinity for endogenous long-chain alcohols and aldehydes, but its main role is participation in the catabolism of formaldehyde, produced after methanol poisoning [31]. ADH IV is the major class taking part in first-pass metabolism of ethanol (FPM). It constitutes a metabolic barrier in stomach against orally receiving alcohol and ethanol produced during bacterial fermentation. Moreover, the activity of ADH IV in retinol metabolism is 6 times higher than for ADH II and 14 times higher than for ADH class I [32]. The role of ADH isoenzymes of higher classes is still unknown.

In humans, there are multiple forms of ALDH divided into two groups: cytoplasmic forms (ALDH I, ALDH III, ALDH VII, ALDH VIII, ALDH IX) and mitochondrial forms (ALDH II, ALDH IV, ALDH V, ALDH VI) [33]. ALDH I is distributed mainly in the liver and catalyzes oxidation of acetaldehyde but also all *trans*- and 9-*cis*-retinal [34]. Of the all isoenzymes, the mitochondrial ALDH II plays a major role in human acetaldehyde metabolism while the others metabolize a variety of substances. ALDH2*2, a genetic polymorphism of ALDH II is prevalent in Asian populations, and these individuals show high blood acetaldehyde concentrations after the intake of only moderate alcohol amount [35]. Class III of ALDH exhibits a high activity in stomach, lungs, liver, skin and the cornea [36]. Recently, ALDH III was found in the saliva, which represents a first barrier against toxic aldehydes present in the food [37]. The other classes of ALDH are distributed widespread in human body but do not participate in acetaldehyde oxidation. These isoenzymes play a role in elimination of toxic aldehydes, which are produced during lipids peroxidation. They also participate in metabolism of bile acids, bioamine, prostaglandin and steroids dehydrogenation [38].

## ADH and ALDH in malignant diseases

The pathophysiological bases of the alterations produced by ethanol start obviously from its metabolism. Accumulating evidences have shown that metabolism of cancer cells differs from that of normal cells. The changes in the activities of ethanol metabolism enzymes may be a great importance and may, among others, influence carcinogenesis. The differences of the ADH, its isoenzymes and ALDH activities between cancer patients and control groups are given in Table 1.

**Table 1:** Activity of ADH and ALDH in cancer diseases

Cancer	Activity											
	ADH I		ADH II		ADH III		ADH IV		ADH total		ALDH	
	Tissue	Serum	Tissue	Serum	Tissue	Serum	Tissue	Serum	Tissue	Serum	Tissue	Serum
Esophagus	–	–	–	–	–	–	↑	↑	↑	↑	–	–
Stomach	–	–	–	–	–	–	↑	↑	↑	↑	–	–
Liver	↑	↑	–	–	–	–	–	–	↑	↑	↑	–
Pancreas	–	–	–	–	↑	↑	–	–	–	↑	–	–
Colorectum	↑	↑	–	–	–	–	–	–	↑	↑	↓	–
Kidney	↑	↑	–	–	–	–	–	–	↑	↑	–	–
Breast	↓	–	–	–	–	–	–	–	–	–	–	–
Endometrium	↑	↑	–	–	–	–	–	–	↑	↑	–	–
Cervix	↑	↑	–	–	–	–	–	–	↑	↑	–	–
Ovary	↑	↑	–	–	–	–	–	–	↑	↑	–	–
Brain	↑	↑	–	–	–	–	–	–	↑	↑	–	–

### Digestive system cancers

Esophagus is the organ where we can see toxic action of ethanol as solution. Ethanol directly causes damage of mucosa which makes easier the penetration of carcinogenic substance. Human esophageal contains three classes of alcohol dehydrogenase—ADH I, ADH III and ADH IV [39]. Studies of Jelski et al. show that class IV of ADH had the highest activity in esophageal cancer cells in comparison with healthy tissue. Moreover, the total activity of ADH was also significantly higher in cancer than in normal mucosa [40]. Besides increase of ADH activity, ALDH activity in esophageal cancer cells did not differ from the healthy tissues. The much higher activity of ADH without increasing ALDH activity suggests that esophageal cancer cells have the higher ability to ethanol oxidizing than to removing acetaldehyde, which may be important factor favorable to carcinogenesis. Among all isoenzymes present in esophageal, class IV reveals the main role in ethanol oxidation so increased activity of this class in cancer cells causes elevated production of acetaldehyde. Moreover, this class of ADH takes part in the metabolism of lipid peroxidation products, which may also intensify carcinogenesis in esophageal. Changes in enzyme activity can be reflected in serum of the patients. It have been stated that in the sera of esophageal cancer patients the total activity of ADH is elevated because of increased activity of its isoenzyme class IV [41]. Probably the reason of increased activity of ADH IV is releasing of this isoenzyme from cancer cells.

Human gastric mucosa contains various isoenzymes of alcohol dehydrogenase: ADH I, ADH III and ADH IV. These isoenzymes compose a metabolic barrier against orally taken alcohol (first-pass metabolism) and also ethanol produced in the course of bacterial fermentation. The comparison of ADH activities in gastric cancer cells showed that the highest activity was exhibited by class IV ADH and the difference in its activity between cancer and healthy tissue was significant. Therefore, the total activity of ADH was also significantly higher in cancer patients than in healthy ones. This suggests that gastric cancer cells have much higher ability to produce acetaldehyde than to remove it. Changes in ADH class IV activity can influence on retinol as well as lipid peroxidation products metabolism and many dietary carcinogens removal. Both disturbances in these processes and increase acetaldehyde production may be a factor intensifying carcinogenesis [42]. In the sera of gastric cancer patients, changes in ADH activity were also certifiable. The total activity of ADH was 40 % higher in patients with gastric cancer than in control group. The activity of class IV ADH was also much elevated (47 %) in cancer patients in comparison with healthy subjects [43]. The increase activities of total ADH and isoenzyme class IV may be explained by its high activity in gastric cancer tissue.

Liver is an organ which is especially exposed for ethanol and its metabolites actions because over 90 % of absorbed ethanol is metabolized in hepatocytes. Human liver contains almost all classes of alcohol dehydrogenase, but the highest activity reveals class I. In hepatocellular carcinoma, the activity of ADH I was more than 26 % higher than in healthy liver. The total activity of ADH was also significantly elevated in cancer in comparison with normal tissue. Moreover, the activity of ADH was much higher than the activity of ALDH (ratio 55:1) which is not enough to prevent acetaldehyde accumulation [44]. In the serum of liver cancer patients, increase of ADH total activity was found. That change was positively correlated with increased activity of class I ADH. It is probably the result of release of ADH I from liver cancer cells or from hepatocytes damaged by the tumor [45].

Colorectal cancer is the next malignant disease in which the activity of ADH and ALDH was studied, and the results are similar to those from hepatocellular carcinoma. The total activity of ADH and the ADH I activity were significantly elevated in colorectal cancer tissue than in healthy mucosa. It is interesting that the activity of ALDH was significantly lower in cancer compared to healthy cells [46]. It leads to very high disproportion in the activities of ADH and ALDH which may result in intensive generation of toxic acetaldehyde. All the more, class I ADH has the highest participation in acetaldehyde production and the activity of this isoenzyme is elevated more than 40 % in colorectal cancer tissue than in normal mucosa [46]. Chronic alcohol consumption decreases retinoic acid production which also can be involved in cell evaluation to premalignant states. One possibility to keep the retinoic acid level normal could be the increased expression of alcohol dehydrogenase isoenzymes. Seitz et al. [47] have showed that in colorectal polyps ADH IV was expressed which is normally not found in the premalignant states. The changes of ADH activity in a tissue are also reflected in the sera. In the course of colorectal cancer, ADH I and the total ADH activity were elevated in the serum of the patients. Moreover, there was a tendency of ADH I activity to increase in accordance with the advancement of cancer and was the highest in the IV stage [48]. The source of that change can be releasing of ADH I from colorectal cancer cells or from liver damaged by metastatic disease.

Ethanol can be metabolized in the pancreas on both the oxidative (ADH, MEOS) and non-oxidative pathways (fatty acid ethyl ester synthetase). The metabolites of these processes: acetaldehyde and fatty acid ethyl esters (FAEEs) are commonly known factors causing pancreas damage and induction of carcinogenesis. The development of pancreas cancer can be also associated with increased activity of class III ADH. Studies show that in the cells of pancreatic cancer, there was significantly elevated activity of ADH isoenzyme III in comparison with healthy tissue [49]. ADH III does not take part in ethanol oxidation but participates mainly in metabolism of endogenous long-chain alcohols and aldehydes. Moreover, this class of ADH catalyzes the oxidation of S-hydroxymethylglutathione. It may lead to depletion of glutathione, which is a strong antioxidant compound responding for maintenance of redox state in cells. The consequence of decreased glutathione concentration may be generation of reactive oxygen species and induction of oxidative stress leading to cancer development. In the sera of pancreatic cancer patients appears the increased activity of total ADH associated with elevated activity of its isoenzyme class III, which may be derived from cancer cells [50].

### Gynecological cancers

The mechanism by which alcohol increases the risk of cancer is associated also with some hormonal changes. The consumption of alcoholic beverages may initiate cancer development by enhancing the level of estrogen in the body and affecting estrogen metabolism. Alcohol could increase plasma estrogen levels either by promoting the induction of aromatases, which can convert androgens to estrogens, or by impairing the metabolism of estrogens in liver, resulting in an accumulation of circulating estrogen [51]. Human uterus contains ADH of class I, III and IV isoenzymes. Study of Orywal et al. has shown that the activity of class I ADH was significantly higher in endometrial cancer cells than in healthy endometrium. Therefore, the total ADH activity was also significantly elevated in cancer tissue in comparison with normal endometrial cells. Moreover, the activity of class I ADH was significantly higher in premenopausal women compared to postmenopausal women, both in cancer and in healthy endometrial cells. The activity of ALDH did not differ between studied groups [52]. A greater capability for ethanol oxidation and less ability to remove acetaldehyde may lead to its accumulation in endometrium, contributing to cancer development. In addition, some reports have shown that the expression of ADH I gene is regulated by sex hormones, which elevated level is noted at women who consume ethanol, what also can be associated with carcinogenesis in uterus [53]. The analyses of ADH isoenzymes activity in the sera of endometrial cancer patients reveal similar correlations. The activities both total ADH and ADH class I were significantly higher in cancer patients compared to healthy women and the patients with myoma uteri [54].

The next women malignant disease in which disturbances in ethanol metabolizing enzymes were stated is the cervical cancer. The activity of class I ADH was significantly higher in cervical cancer cells than in healthy mucosa. The total ADH activity was also elevated in cancer tissue without any changes in ALDH activity. Moreover, the changes of ADH and ALDH were independent from different histological types of cervical cancer. On the other hand, analysis of the particular ADH isoenzymes depending on the progression stage of the cancer showed a tendency toward increased ADH I activity in accordance with the advancement of the disease. The activities of both ADH I and the total ADH were significantly higher in every stage (from I to III) of cancer compared to the controls [55]. The difference of ADH and ALDH activity between cancerous and healthy tissue may cause disorders in the retinol metabolism which can intensify carcinogenesis. This findings are consistent with study of Song et al. [56] who demonstrated that metabolic disturbances of retinal and retinoic acid may be involved in cervical carcinogenesis.

Hormonal factors are involved in the etiology of ovarian cancer, and 60 % of ovarian tumors are estrogen-receptor positive. Alcohol consumption is correlated with endogenous hormone levels. In premenopausal women, alcohol consumption has been consistently, positively correlated with both total estrogen level and bioavailable estrogens, but postmenopausal alcoholics have elevated levels of serum estradiol and plasma levels of estrone sulfate [57, 58]. Similar to findings in the endometrial and cervical cancer, the activity of ADH (especially ADH class I) was significantly higher in ovarian cancer tissue than in ovarian cysts and healthy ovary, but the activity of ALDH was not changed [59]. This suggests increased ability to produce acetaldehyde by cancerous ovarian cells. Acetaldehyde interferes with DNA synthesis and repair causing inhibition in the *O* ^6^-methylguanine-DNA methyltransferase (MGMT) activity, which can be involved in cancer pathogenesis [9]. Moreover, many studies indicate that the retinoic acid pathway plays a role also in ovarian carcinogenesis. Alcohol exposure may lower RA levels either by blocking alcohol dehydrogenase-activated RA biosynthesis due to the competition, as dehydrogenase substrates, between alcohol and vitamin A [16]. Disturbances between ADH and ALDH activities in cancer cells and not-cancerous ovarian tissues can be a factor entangled in ovary carcinogenesis. Besides, cancer cells can release enzymes and change enzymatic profile in the sera of the patients.

### The other cancers

The role of alcohol consumption in the increased incidence rates of renal cancer (RCC) has been inconsistent. Most of the studies provide supportive evidence of a negative association between alcohol intake and risk of RCC, which is associated with the diuretic effect of alcoholic beverages causing reduction in the concentration of carcinogens and decrease in time of its contact with renal epithelial cells [60]. Only one cohort study suggested that intermediate and heavy drinking can be associated with carcinogenesis in kidney [61]. In renal cancer cells, there was a significantly higher activity of total alcohol dehydrogenase and ADH class I compared to healthy kidney; however, the activity of ALDH was not different between both tissues types. Moreover, the activity of total ADH and ADH I isoenzyme was found to be significantly higher already in patients in II stage of renal cancer [62]. These findings suggest increased ability to produce acetaldehyde by cancerous renal cells, already at the beginning of the disease, compared to normal renal tissue, which can cause or intensify carcinogenesis in this organ. The increase in enzyme activity in RCC tissue is reflected by increased activity in sera of cancer patients. The increase in total ADH and ADH I activity in sera of renal cancer patients was positively correlated with increased activity in cancer cells, which suggests that tumor cells can release this enzyme. Moreover, increase in ADH total and ADH I activity in every stage of renal cancer (II–IV) suggests that these enzymes could indicate the presence of the neoplasm. Also the serum activity of ADH total and ADH I tended to be higher in renal cancer patients with a more advanced stage (article in press).

In epidemiological studies, the consumption of alcohol has been identified as a risk factor for breast cancer but the mechanism of this correlation is unclear. One hypothesis is associated with disorders in reproductive steroid hormone concentration caused by ethanol consumption, and the second is related to toxic acetaldehyde affecting damage to epithelial cells of the breast gland [63]. The activity of alcohol and aldehyde dehydrogenase in the breast cancer was also the subject of research of Jelski et al. It is interesting that findings in this type of cancer show inverse association than that which occurs in many malignant diseases. The activity of class I ADH was significantly lower in breast cancer tissue than in healthy parenchyma. Moreover, ADH I activity in the tumor cells were decreasing together with the progression of the disease and in invasive stage was two times lower than in normal mammary tissue [64]. The reduction in the ability of enzymes needed for ethanol detoxification can lead to redox changes and metabolic disorders and contribute to carcinogenesis. Some studies suggest that there is a relationship between an excess of aldehyde products of lipid peroxidation and carcinogenesis of the breast gland so decreased activity of ADH I causes the loss of its protective role in this process [65]. The curious findings refer also to the sera of breast cancer women. The activity of ADH I was significantly higher in the sera of patients only with stage IV breast cancer as compared to healthy controls. However, probably this was a result of isoenzymes being released from organs damaged by metastatic disease, e.g., liver [66].

Alcohol is capable of traversing the blood–brain barrier, and many toxic effects of ethanol are mediated by the acetaldehyde and thus can be a possible risk factor for brain cancer [3]. It is found that alcohol metabolism in brain depends mostly on catalase and cytochrome P450 2E1 and only in 20 % on oxidation with alcohol dehydrogenase [67]. However, in brain cancer tissue the activities of ADH and ADH I were significantly higher than in healthy brain cells and the activity of ALDH was not different between both tissues [68]. The level of acetaldehyde depends on the balance between ADH and ALDH, so these findings suggest that the brain cancer cells have the higher ability to ethanol oxidation and the less capability to removing acetaldehyde. The differences between histological types and the localization of brain cancers were not found [68]. Similar to brain cancer tissue, the activities of total ADH and ADH I were significantly higher in the sera of patients with brain cancer compared to control group with no differences between histological types and cancer localization [69]. Elevated activity in the sera of brain cancer patients of the same isoenzyme where high activity was found in cancer cells seems to be the result of its releasing from tumor cells.

## Summary

Alcohol consumption is a risk factor for many malignant diseases. The exact mechanism of ethanol-associated carcinogenesis has remained unknown. One of the hypotheses is that increased ethanol metabolism leads to elevated production of carcinogenic acetaldehyde. In the cells of many cancers, the elevated activity of alcohol dehydrogenase was found compared to healthy tissue. Moreover, there was a disproportion between activities of ADH and ALDH, which leads to increased ability for ethanol oxidation and less capability to remove acetaldehyde which results in its accumulation and intensification of carcinogenesis. The changes in the activities of particular isoenzymes can have also influence on cancer development because of causing disorders in the metabolism of many biologically important substances. Besides, the cancer cells of many organs may release ADH isoenzymes to the blood which is the reason of elevated activity of the specific isoenzymes in the sera of cancer patients. The significant increase of these isoenzymes in the sera creates the possibility of application ADH activity measurement in the cancer diagnostics.
